# Serotonin receptor 3A controls interneuron migration into the neocortex

**DOI:** 10.1038/ncomms6524

**Published:** 2014-11-20

**Authors:** Sahana Murthy, Mathieu Niquille, Nicolas Hurni, Greta Limoni, Sarah Frazer, Pascal Chameau, Johannes A. van Hooft, Tania Vitalis, Alexandre Dayer

**Affiliations:** 1 Department of Mental Health and Psychiatry, University of Geneva Medical School, CH-1211 Geneva 4, Switzerland; 2 Department of Basic Neurosciences, University of Geneva Medical School, CH-1211 Geneva 4, Switzerland; 3 Swammerdam Institute for Life Sciences, Center for NeuroScience, University of Amsterdam, Sciencepark 904, 1098 XH Amsterdam, The Netherlands; 4 CNRS-UMR 8249, Brain Plasticity Unit, ESPCI ParisTech, 10 rue Vauquelin, 75005 Paris, France

## Abstract

Neuronal excitability has been shown to control the migration and cortical integration of reelin-expressing cortical interneurons (INs) arising from the caudal ganglionic eminence (CGE), supporting the possibility that neurotransmitters could regulate this process. Here we show that the ionotropic serotonin receptor 3A (5-HT_3A_R) is specifically expressed in CGE-derived migrating interneurons and upregulated while they invade the developing cortex. Functional investigations using calcium imaging, electrophysiological recordings and migration assays indicate that CGE-derived INs increase their response to 5-HT_3A_R activation during the late phase of cortical plate invasion. Using genetic loss-of-function approaches and *in vivo* grafts, we further demonstrate that the 5-HT_3A_R is cell autonomously required for the migration and proper positioning of reelin-expressing CGE-derived INs in the neocortex. Our findings reveal a requirement for a serotonin receptor in controlling the migration and laminar positioning of a specific subtype of cortical IN.

## Abstract (web summary)

During brain development, neuronal excitability controls the laminar migration of cortical interneurons from the caudal ganglionic eminences (CGEs). Here the authors identify the 5-HT_3A_ receptor as a specific marker of CGE-derived cortical interneurons (cINs), and as a stimulator of cIN migration.

## Graphical abstract

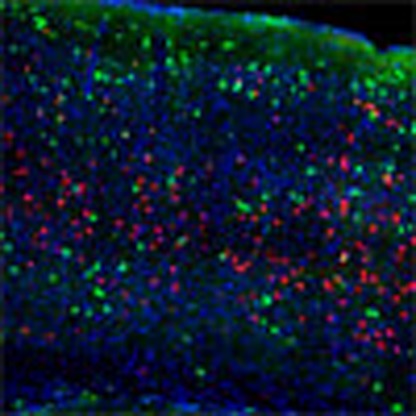

Cortical interneurons (INs) are key cellular components involved in the assembly and function of cortical circuits^1^. Alterations in their development and functional integration are thought to play a critical role in the emergence of psychiatric disorders^2^. In mice, cortical INs mainly originate (about 70%) from the medial ganglionic eminence (MGE), which generates parvalbumin (PV)-expressing basket cells and chandelier cells or somatostatin (SST)-expressing Martinotti cells^3^,^4^,^5^. In addition to the MGE, the caudal ganglionic eminence (CGE)^6^,^7^,^8^,^9^,^10^,^11^,^12^ and to a smaller extent the preoptic area^13^,^14^ contribute to the remaining 30% of cortical INs. CGE-derived interneurons (cINs) are comprised of diverse IN subtypes divided into two main classes: bipolar or double-bouquet INs that express the vaosintestinal peptide (VIP) and neurogliaform or multipolar INs that express reelin and/or neuropeptide Y (NPY)^15^,^16^,^17^.

Recent *in vivo* data have demonstrated that neuronal excitability controls the laminar positioning of reelin-expressing CGE-derived INs^18^. These data support the possibility that cell-extrinsic factors such as neurotransmitters could regulate early activity of migrating cINs and control their final laminar positioning. Interestingly, cINs express the serotonin receptor 3A (5-HT_3A_R) during the process of neuronal migration^15^,^16^. The 5-HT_3A_R is the only known ionotropic serotonergic receptor^19^,^20^ and is expressed exclusively in inhibitory GABAergic INs in the adult cortex^17^,^21^. Serotonin is detected as early as E10.5 in the embryonic forebrain^22^ and has been shown to regulate a variety of developmental processes involved in the construction of cortical circuits^23^, including neuronal migration^24^,^25^ and thalamo-cortical pathfinding^26^. In addition, early-life serotonin dysregulation plays a key role in vulnerability to psychiatric disorders^27^,^28^. Here we determined whether 5-HT_3A_R-dependent signalling controls the migration of cINs.

The migration of INs is a multistep process regulated by the combinatorial expression of transcription factors and by a variety of cell-extrinsic factors including the extracellular matrix and guidance cues^3^,^4^,^5^. To populate the neocortex, INs exit the subpallium and migrate tangentially through migratory streams mainly located in the marginal zone (MZ), subplate and the subventricular zone (SVZ)^2^,^3^,^4^,^29^. Following this initial phase of tangential migration, INs migrate radially and progressively invade the cortical plate (CP)^2^,^3^,^4^,^29^.

To investigate the molecular mechanisms regulating different steps in the migration of cINs, we performed a microarray gene expression analysis on INs preferentially derived from the CGE at three distinct developmental stages of the migratory process. Using this approach, we find that the *Htr3a* is upregulated in INs during the process of CP invasion. Calcium imaging and electrophysiological recordings indicate that the 5-HT_3A_R is functional in cINs during CP invasion and that 5-HT_3A_R activation increases their migratory speed during this late phase of migration. Genetic loss-of-function experiments combined to time-lapse imaging and *in vivo* grafts reveal that the 5-HT_3A_R regulates the entry of cINs into the CP in a cell-autonomous manner. Finally, we find that genetic deletion of the 5-HT_3A_R led to the persistent laminar mispositioning of reelin-expressing CGE-derived INs.

## Results

### Migrating cINs specifically express functional 5-HT_3A_R

**Figure 1:**
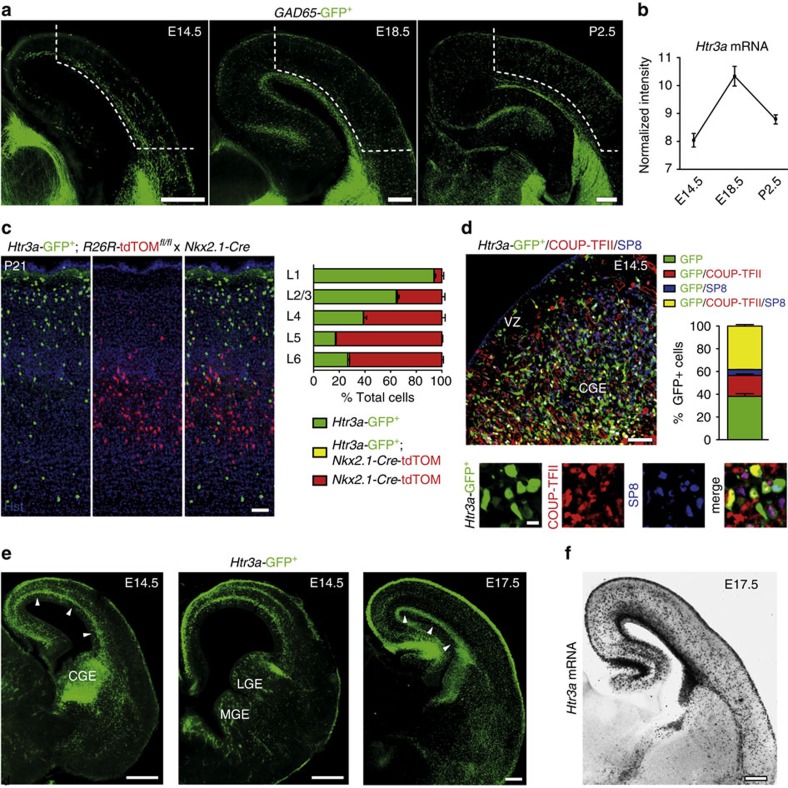
5-HT_3A_R is specifically expressed and upregulated in caudal ganglionic eminence (CGE)-derived interneurons (INs) during the phase of cortical invasion. (**a**) Microdissection of cortical tissue (dotted lines) containing *GAD65*-GFP^+^ INs was performed at three developmental time points corresponding to the phase of tangential migration (E14.5), cortical invasion (E18.5) and termination of migration (P2.5). *GAD65*-GFP^+^ INs were isolated using fluorescence-assisted cell sorting (FACS) and gene expression analysis was performed using microarrays. (**b**) Microarrays revealed that *Htr3a* mRNA expression increases during cortical invasion in FACS-isolated *GAD65*-GFP^+^ INs (*n*=3 replicates at each time point). (**c**) Genetic fate mapping indicates that *Htr3a-*GFP^+^ INs only rarely (<%1) overlap with *Nkx2.1-*Cre; TOM^+^ cells and preferentially populate superficial cortical layers at P21 (*n*=4,636 *Htr3a*-GFP^+^ cells and 7,588 TOM^+^ cells in four brains). (**d**) The majority of *Htr3a-*GFP^+^ INs located in the mantle zone of the CGE at E14.5. CGE cells are immunolabelled for the CGE-enriched transcription factors COUP-TFII and/or SP8 (*n*=3,451 *Htr3a*-GFP^+^ cells in three brains). (**e**) *Htr3a-*GFP^+^ INs populate the CGE but not the medial ganglionic eminence (MGE) and migrate tangentially to reach the dorsal pallium through the subventricular zone (SVZ) stream (arrowheads). (**f**) *In situ* hybridization showing that the expression pattern of the *Htr3a* mRNA at E17.5 is similar to *Htr3a-*GFP^+^ mouse. Error bars are means±s.e.m. COUP-TFII, chicken ovalbumin upstream promoter transcription factor 2; Hst, Hoechst; LGE, lateral ganglionic eminence; VZ, ventricular zone. Scale bars: (**a**) 200 μm; (**c**) 50 μm; (**d**) 50 μm (low magnification), 10 μm (high magnification); (**e**,**f**) 200 μm.

To study the migration of CGE-derived INs, we used *GAD65*-green fluorescent protein (GFP) mice that preferentially label CGE-derived INs and rarely MGE-derived INs expressing PV and SST (<5% at P21)^30^,^31^ (Supplementary Fig. 1). To identify time-specific genes regulating the phase of cortical invasion, microarray-based gene expression analysis of *GAD65*-GFP^+^ INs was performed at three different developmental time points corresponding to distinct steps of the migratory process (Fig. 1a). *GAD65*-GFP^+^ cells in the process of tangential migration were obtained by microdissection of the E14.5 caudal pallium containing the SVZ migratory stream. *GAD65*-GFP^+^ cells invading the cortex were obtained by microdissection of the developing cortex at E18.5 (excluding the SVZ), whereas INs terminating migration were obtained by microdissection of the cortex at P2.5 (excluding the SVZ). *GAD65*-GFP^+^ INs were then isolated using fluorescent-assisted cell sorting (FACS; Supplementary Fig. 2a,b), total RNA was extracted and microarrays were performed. Data were analysed in order to identify genes specifically upregulated during cortical invasion, that is, genes that significantly increased their expression from E14.5 to E18.5 (*P*<0.01 and >2-fold change) and significantly decreased their expression from E18.5 to P2.5 (*P*<0.01 and >2-fold change). Using this unbiased approach, we found that *Htr3a* is the third top candidate gene, displaying a 4.9-fold upregulation from E14.5 to E18.5 (Fig. 1b, Supplementary Table 1). Genetic fate mapping indicated that cINs expressing the 5-HT_3A_R do not originate from MGE-derived progenitors that specifically express the transcription factor *Nkx2.1* (ref. 32). After crossing *Nkx2.1*-Cre mice with *Htr3a-*GFP; *R26R-*tdTOM^fl/fl^ reporter mice, we observed that *Htr3a-*GFP^+^ INs only rarely overlapped with TOM^+^ cells at E17.5, P0.5, P21 and preferentially populated superficial cortical layers (Fig. 1c, Supplementary Fig. 2c,d). In line with these results, we observed that at E14.5, *Htr3a-*GFP^+^ cells were mainly found populating the CGE and not the MGE (Fig. 1d, e, Supplementary Fig. 2f). In the CGE, the majority of *Htr3a-*GFP^+^ cells expressed COUP-TFII and/or SP8 (Fig. 1d), two transcription factors preferentially expressed in this region during the embryonic period^33^,^34^ and only very rarely NKX2.1 (Supplementary Fig. 2e). *Htr3a-*GFP^+^ INs were observed at E14.5 exiting the subpallium by forming a tangential stream of migratory INs located in the pallial SVZ and invading the CP by E17.5 (Fig. 1e). *In situ* hybridization targeting the *Htr3a* mRNA confirmed the labelling pattern observed in *Htr3a-*GFP^+^ brains at E17.5 (Fig. 1f) and revealed strong expression in the CGE but not MGE at E14.5 (Supplementary Fig. 2f).

To determine whether the 5-HT_3A_R is functional in migrating cINs, we isolated the CGE from E14.5 *Htr3a*-GFP slices and platted cINs *in vitro* in the presence of dissociated and isochronic cortical cells in order to allow *Htr3a*-GFP^+^ INs to migrate on a homogeneous substrate^35^ (Supplementary Fig. 3a). Calcium imaging revealed that application of the 5-HT_3A_R agonists *m*-chlorophenylbiguanide (*m*CPBG) or SR57227 could repeatedly induce calcium events in migrating *Htr3a*-GFP^+^ INs and this could be observed in the presence of the sodium channel blocker tetrodotoxin (TTX), the NMDA (*N*-methyl-D-aspartate) antagonist D(-)-2-amino-5-phosphonovaleric acid (D-AP5), the AMPA/kainate antagonist 2,3-Dioxo-6-nitro-1,2,3,4-tetrahydrobenzo[f]quinoxaline-7-sulfonamide (NBQX) and the GABA-A antagonist gabazine (Supplementary Fig. 3b–e). Furthermore, a significant time-dependent increase in 5-HT_3A_R-induced calcium transients was observed in migrating cINs from E14.5 day *in vitro* 1 (+DIV1) to E14.5 (+DIV3) and this was linked to a significant increase in protein expression of the 5-HT_3A_R (Supplementary Fig. 3f,g). To demonstrate that 5-HT_3A_R on cINs are directly activated in acute cortical slices, electrophysiological recordings were performed in *Htr3a*-GFP^+^ INs. Recordings revealed that 5-HT_3A_R activation directly triggers inward currents in migrating cINs in the CP of cortical slices and this was observed in the presence of TTX, D(-)-2-amino-5-phosphonovaleric acid, NBQX and gabazine (Supplementary Fig. 4 and Supplementary Table 2). Interestingly, 5-HT_3A_R-mediated inward currents were detected in *Htr3a-*GFP^+^ INs migrating in the E18-E19 CP but not in cINs migrating tangentially in the SVZ at E18-E19 or IZ/SVZ at E14-E15 (Supplementary Fig. 4 and Supplementary Table 2), indicating that *Htr3a-*GFP^+^ INs migrating in the CP become functionally responsive to 5-HT_3A_R activation. At P2.5, 5-HT_3A_R activation reliably induced currents in *Htr3a*-GFP^+^ cells located in the CP, suggesting that although *Htr3a* mRNA is downregulated in cINs from E18.5 to P2.5 targeting of the 5-HT_3A_R to the cell membrane may increase through post-translational modifications^20^. Taken together, these data confirm previous findings^15^,^16^, indicating that functional 5-HT_3A_R is specifically expressed in CGE-derived but not MGE-derived INs. Furthermore, functional upregulation of 5-HT_3A_R in cINs during the process of cortical invasion suggests that 5-HT_3A_R could regulate this migratory phase.

### 5-HT_3A_R activation stimulates the migration of cINs

**Figure 2:**
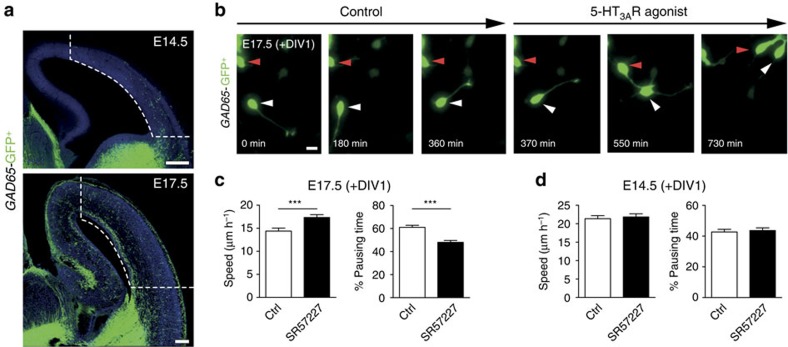
5-HT_3A_R activation increases the migratory speed of CGE-derived interneurons (INs) during the phase of cortical plate invasion. (**a**) Microdissection of cortical tissue (dotted lines) containing *GAD65*-GFP^+^ INs was performed at E14.5 corresponding to the phase of tangential migration or at E17.5 during the phase of cortical invasion. *GAD65-GFP*^+^ INs were platted in culture and time-lapse imaging was performed at day *in vitro* 1 (+DIV1). (**b**) Illustrative time-lapse sequence showing that *GAD65*-GFP^+^ INs (arrowheads) at E17.5 (+DIV1) increase their migration after exposure to the 5-HT_3A_R agonist SR57227 (100 nM). Cells were tracked during a control period and a drug period of 360 min each. (**c**, **d**) Quantification revealed that 5-HT_3A_R activation significantly increases the migratory speed and decreases the pausing time of *GAD65*-GFP^+^ INs at E17.5 (+DIV1; *n*=156 cells in three independent experiments; **c**) but not at E14.5 (+DIV1; *n*=140 cells in three independent experiments; **d**). ***P<0.001, paired Student’s *t*-test. Error bars are means±s.e.m. Scale bars: (**a**) 150 μm; (**b**) 10 μm.

To determine whether 5-HT_3A_R activation could modulate the migratory behaviour of cINs, *GAD65*-GFP^+^ INs in the E14.5 SVZ/IZ stream or invading the E17.5 cortex were isolated through microdissection and time-lapse imaging of INs was performed in cortical cultures at E14.5 (+DIV1) and E17.5 (+DIV1) in a serotonin-free medium^26^ (Fig. 2a,b). This *in vitro* strategy has successfully been used to unmask the effects of GABA-A stimulation on cortical IN migration^35^. Furthermore, it has the advantage to allow the recordings of INs in a serotonin-free environment, thus removing the potential confounding effects of endogenous levels of serotonin contained in acute slices. Indeed, it has been shown that it is not possible in cortical slices to unmask the role of 5-HT_3A_R on the migration of cINs^25^. Using this *in vitro* strategy, time-lapse recordings revealed that the 5-HT_3A_R agonist SR57227 but not vehicle exposure significantly increased the mean migratory speed and significantly decreased the pausing time of *GAD65*-GFP^+^ INs at E17.5 (+DIV1; Fig. 2c, Supplementary Fig. 5a, Supplementary Movie 1). In contrast, 5-HT_3A_R-stimulation did not affect the migratory speed or pausing time of cINs at E14.5 (+DIV1; Fig. 2d, Supplementary Fig. 5b, Supplementary Movie 2). Given that it has been reported that high concentrations of serotonin (200–400 μM) inhibit cIN migration possibly through a 5-HT_6_R-mediated mechanism^25^, we aimed to determine whether more physiological concentrations of serotonin (nanomolar range) could have a pro-migratory effect and mimic the effects of 5-HT_3A_R-stimulation. Indeed, exposure to serotonin (100 nM) significantly increased the mean migratory speed and significantly decreased the pausing time of *GAD65*-GFP^+^ INs at E17.5 (+DIV1) but not at E14.5 (+DIV1; Supplementary Fig. 5c–f). Taken together, these functional investigations indicate that 5-HT_3A_R activation increases the migratory speed of CGE-derived INs during the phase of CP invasion.

### 5-HT_3A_R specifically regulates growth cone dynamics of cINs

**Figure 3:**
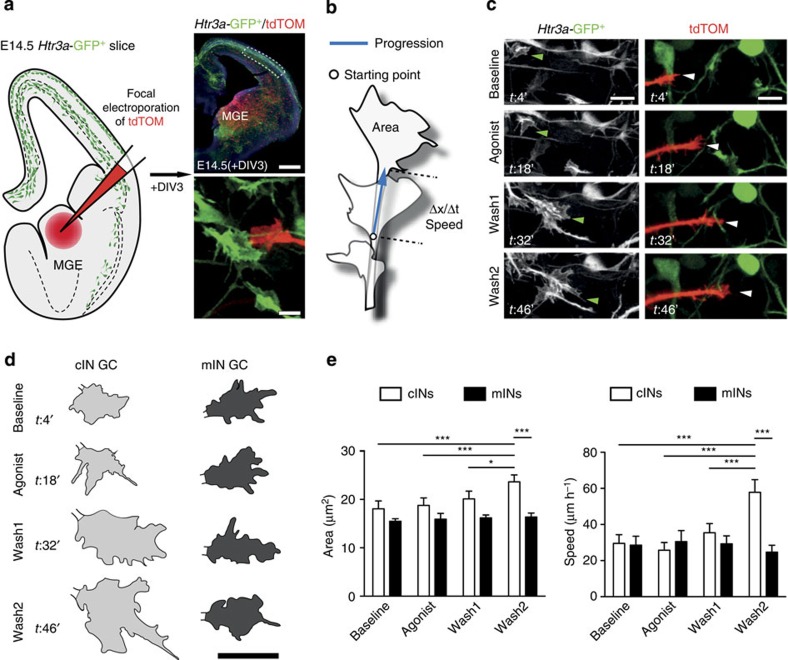
The 5-HT_3A_R specifically regulates growth cone dynamics of interneurons (INs) derived from the CGE but not the MGE. (**a**) Schema illustrating that MGE-derived INs (mINs) were labelled using focal electroporation of a tdTOM-expressing plasmid in the MGE, whereas CGE-derived INs (cINs) were labelled using *Htr3a-*GFP^+^ slices. At E14.5 (+DIV3), confocal time-lapse imaging of the growth cones (GCs) of cINs and mINs was performed in the cortical plate (dotted area) and GC dynamics was assessed at baseline, during and after 5-HT_3A_R activation with *m*-chlorophenylbiguanide (*m*CPBG 100 μM). (**b**) Growth cone dynamics was assessed by tracing the GC area in sequential time-lapse images and by calculating the speed of progression of the growth cone. (**c**) Time-lapse sequence illustrating that 5-HT_3A_R activation induces a delayed increase in the GC size of *Htr3a*-GFP^+^ cINs (green arrowheads) but not TOM^+^ mINs (white arrowheads). (**d**) Illustrative tracing of growth cone areas of *Htr3a-*GFP^+^ cIN and TOM^+^ mIN during the baseline period, during exposure to *m*CPBG and during the two wash periods. Note that GC size of cIN increases during the second wash period but not the GC of mIN. (**e**) Quantification revealed that the GC area and speed of cINs (*n*=13 GC in 10 slices) but not of mINs (*n*=12 GC in 10 slices) significantly increases after 5-HT_3A_R activation (***P<0.001, *P<0.05, two-way analysis of variance with Bonferroni’s test). Error bars are means±s.e.m. Scale bars: (**a**) 200 μm (low magnification), 5 μm (high magnification); (**c**) 10 μm; (**d**) 5 μm.

Growth cones located at the tip of the leading processes of migrating INs play a key role in sensing the local environment. To determine whether 5-HT_3A_R growth cone responsiveness was specific to cINs migrating in the CP, we performed simultaneous time-lapse imaging of the growth cones of MGE-derived INs (mINs) and cINs as they invade the CP (Fig. 3a). MGE-derived INs were labelled by focal electroporation of a Tomato-expressing plasmid in the MGE of *Htr3a-*GFP^+^ cortical slices at E14.5 and growth cone imaging of cINs and mINs was simultaneously performed in the CP at E17.5 (+DIV3) in serotonin-free medium (Fig. 3a–c). Using this paradigm, we found that the size and growth cone extension speed of cINs were specifically increased after *m*CPBG exposure, whereas no effect of the drug was observed in mINs (Fig. 3d,e, Supplementary Movies 3 and 4). To confirm the specificity of the 5-HT_3A_R agonist, similar experiments were performed on the growth cones of *Htr3a*-ko; *GAD65*-GFP^+^ INs. Quantification revealed that 5-HT_3A_R activation failed to increase the GC speed and size of *Htr3a*-ko; *GAD65*-GFP^+^ INs (Supplementary Fig. 5g,h). Taken together, these data indicate that the 5-HT_3A_R specifically regulates the growth cone dynamics of cINs but not mINs during CP invasion.

### 5-HT_3A_R specifically controls the migration of cINs

**Figure 4:**
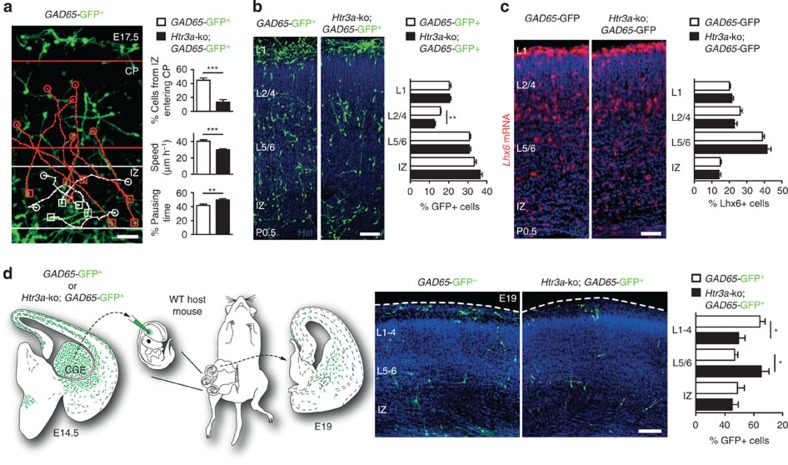
5-HT_3A_R controls the migration and positioning of CGE-derived INs in the cortical plate (CP). (**a**) Examples of migratory paths in E17.5 acute cortical slices showing cINs remaining in the intermediate zone (IZ; white tracks) or invading the CP (red tracks). Squares indicate start positions and circles indicate end positions of tracked cells after 8 h imaging. Quantification showing a significant reduction in the percentage of *Htr3a*-ko*; GAD65*-GFP^+^ INs (*n*=124 cells in five slices) migrating from the IZ into the CP in comparison with *GAD65*-GFP^+^ INs (*n*=106 cells in five slices). In addition, the migratory speed of *Htr3a*-ko; *GAD65*-GFP^+^ INs is significantly decreased, whereas their pausing time significantly increased versus *GAD65*-GFP^+^ INs (***P<0.001, **P<0.01, unpaired Student’s *t*-test). (**b**) *In vivo* quantification reveals significant decrease in the percentage of *Htr3a*-ko; *GAD65*-GFP^+^ INs (*n*=6144 cells in four brains) in layer 2–4 versus *GAD65*-GFP^+^ INs (*n*=6708 cells in four brains; **P<0.01, unpaired Student’s *t*-test) in the prospective P0.5 somatosensory cortex. (**c**) Quantification of MGE-derived INs in the prospective P0.5 somatosensory cortex labelled by *in situ* hybridization for the MGE-specific transcription factor *Lhx6* reveals no significant layering differences between *Htr3a*-ko; *GAD65*-GFP^+^ mice (*n*=2,013 cells in three brains) versus control *GAD65*-GFP^+^ (*n*=3,731 cells in four brains). (**d**) *In vivo* quantification in host E19.0 wild-type (WT) cortex reveals significant misdistribution of isochronic-grafted *Htr3a*-ko; *GAD65*-GFP^+^ INs (*n*=882 cells in five brains) in the developing cortex versus *GAD65*-GFP^+^ INs (*n*=736 cells in four brains; *P<0.05, unpaired Student’s *t*-test). Hst, Hoechst; MZ, marginal zone. Error bars are means±s.e.m. Scale bars: (**a**) 50 μm; (**b**–**d**) 100 μm.

To study the role of the 5-HT_3A_R on the migration of cINs during cortical invasion, time-lapse imaging was performed on E17.5 cortical slices during 10–12 h and single-cell tracking was performed on *GAD65*-GFP^+^ INs and *Htr3a-*ko; *GAD65*-GFP^+^ INs. Quantification revealed that the proportion of *Htr3a-*ko; *GAD65*-GFP^+^ INs migrating from the IZ into the cortex was significantly decreased versus *GAD65*-GFP^+^ INs (Fig. 4a, Supplementary Movies 5 and 6). In addition, the migratory speed of *Htr3a-*ko; *GAD65*-GFP^+^ INs invading the cortex from the IZ was significantly decreased and their pausing time significantly increased compared with *GAD65*-GFP^+^ INs (Fig. 4a). To determine whether decreased cortical invasion of *Htr3a-*ko; *GAD65*-GFP^+^ INs resulted in early positioning defects in the developing cortex, the distribution of *GAD65*-GFP^+^ INs was analysed at birth. Given that CGE-derived INs preferentially target superficial cortical layers (L1-4) in contrast to deep layers L5/6 (Fig. 1c, Supplementary Fig. 2), the distribution of *GAD65*-GFP^+^ INs and *Htr3a-*ko; *GAD65*-GFP^+^ INs were analysed in prospective superficial cortical layers (L1 and L2-4) and deep cortical layers (L5/6) of the developing cortex. Quantification indicated that the percentage of *Htr3a-*ko; *GAD65*-GFP^+^ INs located in superficial layers 2–4 at P0.5 was significantly decreased compared with *GAD65*-GFP^+^ INs (Fig. 4b) and no changes were observed in other compartments, suggesting that the 5-HT_3A_R regulates the positioning of CGE-derived INs in cortical layers to which they are preferentially targeted. The 5-HT_3A_R specifically controlled the migration of cINs, as the distribution of mINs expressing the MGE-specific transcription factor *Lhx6* was unchanged across different cortical layers (Fig. 4c). The distribution of Lhx6^+^ and *GAD65*-GFP^+^ INs in layers 2–4, layer 5/6 and intermediate zone were significantly different at P0.5, indicating that mINs and cINs are in the process of being differentially allocated to specific cortical layers already at birth (Supplementary Fig. 5i). Isochronic graft experiments indicated that 5-HT_3A_R regulates cortical invasion in a cell-autonomous manner. Indeed, at E19, the cortical distribution of E14.5 CGE-isolated *Htr3a-*ko; *GAD65*-GFP^+^ INs grafted into the CGE of wild-type E14.5 embryos was significantly altered in comparison with grafted *GAD65*-GFP^+^ INs (Fig. 4d). Taken together, these results indicate that the 5-HT_3A_R controls the migration and positioning of cINs into the developing cortex.

### 5-HT_3A_R is required for the laminar positioning of cINs

**Figure 5:**
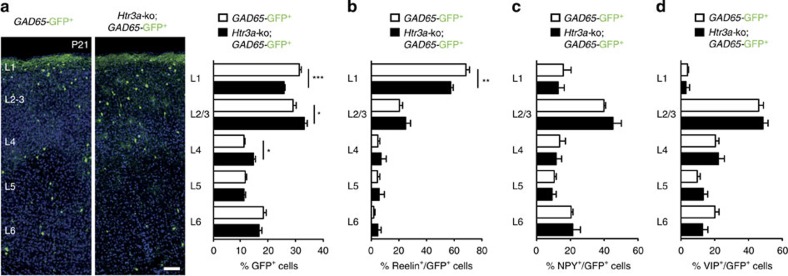
5-HT_3A_R controls the laminar positioning of reelin-expressing CGE-derived INs in the cortex. (**a**) At P21, the positioning of *Htr3a*-ko; *GAD65*-GFP^+^ INs (*n*=2,775 cells in six brains) was significantly altered in superficial layers compared with *GAD65*-GFP^+^ INs (*n*=2,707 cells in six brains; ***P<0.001, *P<0.05 unpaired Student’s *t*-test). (**b**) The percentage of *Htr3a*-ko*; GAD65*-GFP^+^ INs expressing reelin (*n*=706 cells in five brains) was significantly decreased in layer 1 compared with reelin^+^/*GAD65*-GFP^+^ INs (*n*=1,376 cells in six brains; **P<0.01 unpaired Student’s *t*-test). (**c**,**d**) The laminar distribution of *Htr3a*-ko; *GAD65*-GFP^+^ INs expressing NPY (*n*=1,093 cells in eight brains) or VIP (*n*=963 cells in six brains) was not altered compared with NPY^+^ (*n*=1,127 cells in six brains) or VIP^+^/*GAD65*-GFP^+^ INs (*n*=1,219 cells in six brains; unpaired Student’s *t*-test). Error bars are means±s.e.m. NPY, neuropeptide Y; VIP, vasointestinal peptide. Scale bar, (**a**) 50 μm.

To determine whether 5-HT_3A_R deletion leads to persistent alterations in the distribution of cINs, the laminar positioning of CGE-derived INs was analysed in the somatosensory cortex of *Htr3a-*ko mice at P21. Quantification revealed a significant decrease in the percentage of *Htr3a-*ko; *GAD65*-GFP^+^ INs at P21 in superficial layers of the somatosensory cortex compared with *GAD65*-GFP^+^ INs (Fig. 5a). To determine whether the positioning of a specific subtype of CGE-derived INs was altered in *Htr3a-*ko mice, *GAD65*-GFP^+^ sections were stained for reelin, VIP and NPY, which label different CGE-derived IN populations. Quantification revealed that 5-HT_3A_R deletion leads to the persistent laminar mispositioning of CGE-derived reelin^+^ but not VIP^+^ or NPY^+^/*Htr3a-*ko; GAD65-GFP^+^ INs in superficial cortical layers as compared with *GAD65*-GFP^+^ INs (Fig. 5b–d). Taken together, these data indicate that the 5-HT_3A_R regulates the laminar positioning of a specific subclass of reelin-expressing CGE-derived INs.

## Discussion

Here we report that the 5-HT_3A_R, a specific marker of CGE-derived cortical INs, is functionally upregulated in cINs during the process of cortical invasion and that 5-HT_3A_R activation stimulates the migration of cINs during the phase of cortical invasion. Furthermore, 5-HT_3A_R-mediated activation specifically regulated the growth cones dynamic of cINs but not of mINs. Time-lapse imaging, graft experiments and *in vivo* quantification in *Htr3a-*ko mice demonstrated that the 5-HT_3A_R is cell autonomously required for positioning of cINs into the nascent cortex. Finally, alterations in the early process of CP invasion lead to a persistent mispositioning of the reelin-expressing cINs subtype in superficial cortical layers of *Htr3a-*ko mice.

The migration of cINs into the cortex is a multi-step process. After exiting the subpallium, INs disperse into the pallium through specific tangential migratory streams mainly located in the MZ, subplate and SVZ^3^,^4^,^5^,^29^. The selection of migratory streams by mINs requires responsiveness to the chemokine Cxcl12 through the G-protein-coupled receptors Cxcr4 and Cxcr7 (refs 36, 37, 38, 39). It has been hypothesized that the loss of responsiveness to Cxcl12 could allow mINs to become responsive to a yet unknown chemoattractant located in the CP. The chemoattractant properties of the CP have not been elucidated and it is unknown whether cINs are responsive to the same set of guidance cues as mINs. The fact that the 5-HT_3A_R is required for proper CP invasion and that serotonin levels increase during the late phase of cortical invasion^22^ suggests that serotonin signalling through the 5-HT_3A_R could specifically regulate the responsiveness of cINs to guidance cues located in the developing cortex.

Serotonin has been reported to regulate the migration of different non-neuronal cell types including eosinophils, pulmonary artery smooth muscle cells, aortic endothelial cells and cranial neural crest cells^40^,^41^,^42^,^43^,^44^. Depending on the concentration of serotonin, cell types, experimental paradigms and embryonic age of the cells, serotonin was shown to either stimulate or inhibit migration. For example, differential effects of serotonin on the migration of neural crest cells were observed depending on the concentration of serotonin and embryonic age of the cells^41^. Here we report that serotonin levels at the nanomolar range increase the migration speed of cINs in contrast to high levels of serotonin (micromolar range), which have previously been shown to inhibit migration possibly through at 5-HT_6_R mechanism^25^. A requirement for serotonin in regulating neuronal embryonic migration *in vivo* was first described in the nematode *Caenorhabditis elegans*^45^. In mice, time-lapse imaging on cortical slices revealed that an excess of serotonin decreased the migratory speed of INs and pyramidal neurons in a reversible manner^24^,^25^. Interestingly alterations in the positioning of cINs in the developing cortex *in vivo* were reported in a pharmacological model of serotonin depletion^46^ as well as in a genetic model of serotonin excess^25^. During the phase of cortical invasion, serotoninergic raphe fibers located in the intermediate zone and in the MZ^47^ are the main source of serotonin to the developing cortex^26^. Our current model proposes that following the switch from tangential to radial migration cINs increase their responsiveness to serotonin signalling through 5-HT_3A_R upregulation in order to respond to guidances cues located in the developing cortex. In a related developmental process, that is, embryonic thalamo-cortical wiring, a role for serotonin in modulating growth cone responsiveness to the guidance cue netrin1 has been described^26^. In migrating cINs, we find that the effect of 5-HT_3A_R activation on growth cones dynamics is delayed, suggesting that 5-HT_3A_R activation may have indirect effects on growth cone dynamics by modulating responsiveness to guidance cues. Although the guidance cues regulating the migration of cINs into the CP remains to be discovered, the identification of genes specifically upregulated in cINs during CP invasion provides a set of candidate genes that may specifically regulate this process. Among candidate genes from our microarray, *PlexinA4* displayed a time-specific twofold upregulation during CP invasion, suggesting that this receptor could play a role in regulating CP invasion of cINs. Interestingly, PlexinA4/Semaphorin6A signalling was shown to regulate the migration of oligodendrocyte precursor cells in the developing cortex^48^. Further studies are required to establish whether *PlexinA4* expression is regulated by 5-HT_3A_R signalling and regulates the migration of cINs.

The 5-HT_3A_R is a cation-selective ligand-gated ion channel that mediates neuronal depolarization and excitation^20^. Previous work in the field has demonstrated that neuronal excitability is required for the proper positioning of CGE-derived INs in superficial cortical layers^18^, supporting the possibility that neurotransmitters such as serotonin could regulate the migration of CGE-derived INs through 5-HT_3A_R-dependent signalling. Here we show that 5-HT_3A_R activation enhances the migratory speed and growth cone dynamics of cINs during the phase of CP invasion. The 5-HT_3A_R is closely related to the anion selective GABA_A_ receptor. In conditions of low-expression levels of the potassium-chloride co-transporter KCC2, GABA_A_ stimulation induces an increase in calcium transients and promotes the migration of cINs and their entry into the cortex^35^,^49^. In addition, other neurotransmitter systems have been shown to regulate the migration of cortical INs such as AMPA and NMDA receptors^50^,^51^,^52^. Overall, *in vitro* data obtained in cINs but also in other neuronal cell types such as cerebellar granule cells indicate that neuronal migration involves the modulation of calcium transients through the opening of NMDA and voltage-gated calcium channels^35^,^53^,^54^,^55^. It is thus hypothesized that early activity in neuronal networks generates ambient levels of neurotransmitters that regulate IN migration by modulating their intracellular calcium transients. Our data add to this model the possibility that ambient levels of serotonin could specifically regulate the migration of cINs into the CP through the regulation of 5-HT_3A_R-dependent calcium transients.

Developmental insults including alterations in the migration and maturation of cINs contribute to the emergence of psychiatric-relevant functional alterations later in life^2^. Early-life serotonin dysregulation in rodents following genetic deletion or pharmacological blockade of the serotonin transporter by antidepressants leads to long-term psychiatric-related phenotypes^27^. In this perspective, the 5HT_3A_R represents a novel developmental target that regulates several cellular events involved in neural circuit formation. Here we reveal that the 5-HT_3A_R is required to control the migration and positioning of cINs into the CP. In addition to its importance in regulating cIN subtypes, the 5HT_3A_R is expressed in Cajal-Retzius cells and regulates the apical dendritic growth of pyramidal neurons in the cortex through a reelin-dependent mechanism^56^. Taken together, our study provides further insights on the importance of the 5-HT_3A_R in controlling developmental processes involved in the formation of cortical circuits. It proposes that 5-HT_3A_R-mediated signalling constitutes a molecular mechanism controlling the migration and laminar positioning of a reelin-expressing CGE-derived IN subtype and thus further supports the important role of early activity in the development of reelin-expressing CGE-derived INs^18^.

## Methods

### Mice

Animal experiments were conducted according to the Swiss and international guidelines and approved by the local Geneva animal care committee. Adult timed pregnant mice were obtained by overnight (ON) mating and the following morning was counted as embryonic day (E) E0.5. To study CGE-derived INs, we used transgenic mice expressing GFP under the control of the *GAD65* regulatory sequences (*GAD65-*GFP)^25^,^30^ and transgenic mice expressing the enhanced GFP under the control of the *Htr3a* regulatory sequences (*Htr3a-*GFP) provided by the GENSAT Consortium^16^. Both strains were maintained on a C57Bl/6 background. C57Bl/6 wild-type mice were used for *in vivo* graft experiments. The *Nkx2.1*-Cre mice have been previously described^32^ and the *R26R-*tdTOM^fl/fl^ reporter mice were obtained from Jackson Laboratory. *Htr3a-*GFP mice were crossed to *R26R-*tdTOM^fl/fl^ mice to obtain *Htr3a-*GFP; *R26R-*tdTOM^fl/fl^ mice. *Htr3a*-ko mice have been previously described^57^ and crossed to *GAD65-*GFP mice to obtain *Htr3a*-ko; *GAD65-*GFP animals.

### Tissue processing and immunohistochemistry (IHC)

Pregnant females were euthanized by lethal intraperitoneal (i.p.) injection of pentobarbital (50 mg kg^−1^), embryos were collected by caesarian cut and brains were dissected and fixed ON in cold 4% paraformaldehyde (PFA) dissolved in 0.1 M phosphate buffer, pH 7.4. For postnatal brains, animals were deeply anaesthetized by i.p. injection of pentobarbital, transcardially perfused with 0.9% saline followed by cold 4% PFA and postfixed (ON) in cold 4% PFA. Brains were cut on a Vibratome (Leica, VT1000S) for IHC and for free-floating *in situ* hybridization. Sections were kept at 4 °C in 0.1 M phosphate buffer saline and were stained by IHC as described^31^ with the following primary antibodies rabbit anti-GFP (1:500; Millipore), goat anti-GFP (1:1,000; Chemicon), mouse anti-human ovalbumin upstream promoter transcription factor 2 (COUP-TFII; 1:500; Perseus proteomics), goat anti-SP8 (1:50; Santa-Cruz), rabbit anti-NKX2.1 (1:100; Santa-Cruz), mouse anti-PV (1:1,000; Swant), rat anti- SST (1:500; Millipore), mouse anti-Reelin (1:500; Abcam), rabbit anti-VIP (1:500; Abcam), rabbit anti-NPY (1:1,000; Immunostar), rabbit anti-Calretinin (1:1,000; Swant). Secondary goat or donkey Alexa-488, -568 and -647 antibodies (Molecular Probes, Invitrogen) raised against the appropriate species were used at a dilution of 1:500–1,000 and sections were counterstained with Hoechst 33258 (1:10,000).

### *In situ* hybridization

Sections were hybridized as described previously^25^ with the respective DIG-labelled RNA probes. The *Htr3a* plasmid probe^16^ was linearized with *HindIII-HF* for RNA probe synthesis by T7 polymerase (kind gift from Dr B. Emerit). The *Lhx6* plasmid probe^58^ (kind gift from Dr M. Denaxa) was linearized with *Not*1 for antisense RNA probe synthesis by T3 polymerase. The unbound probe was washed and slices were incubated with alkaline phosphatase-conjugated anti-DIG antibody (1:2,000; Roche) ON at 4 °C. NBT/BCIP (Roche) was then used as an alkaline phosphatase substrate to reveal the hybridized probe. Fast Red (Roche) was used as an alkaline phosphatase fluorescent substrate.

### Preparation of dissociated cultures and acute slices

For migration studies, E14.5 and E17.5 cortices from *GAD65*-GFP^+^ coronal sections were isolated under a fluorescence scope (Leica, M165 FC) through microdissection. For calcium recordings, microdissection of the CGE of E14.5 *Htr3a-*GFP caudal coronal slices and cortices of E14.5 wild-type coronal slices was performed. Isolated tissue was collected in Hanks’ balanced salt solution (HBSS), manually triturated and dissociated in trypsin at 37 °C for 15 min and blocked in fetal calf serum. Dissociated cells were then centrifuged for 5 min at 1,200 r.p.m. and pellets were resuspended in neurobasal medium (NBM; Invitrogen) supplemented with 2% B27 (Gibco), 2 mM glutamine, 1% penicillin–streptomycin, 2 mM *N*-acetyl-cysteine and 1 mM sodium pyruvate. For migration assays, cells were plated in 96-well microplates (Costar 3720) coated with laminin (10 μg ml^−1^; Invitrogen) and poly-D-lysin (0.1 mg ml^−1^; Sigma). Acute brain slices were prepared as described^24^ from *Htr3a*-GFP+, *GAD65*-GFP+ and *Htr3a*-ko; *GAD65*-GFP^+^ embryos. Briefly, brains were extracted, embedded in HBSS with 3% ultra-pure low-melting point agarose (Invitrogen or Roth) and 250-μm-thick slices were cut on a Vibratome (VT1000S; Leica) in cold oxygenated (95% O_2_, 5% CO_2_) artificial cerebrospinal fluid (ACSF) containing (in mM): NaCl (125), KCl (3), CaCl_2_ (1.6), MgCl_2_ (1.5), NaH_2_PO_4_ (1.25), NaHCO_3_ (26), D-glucose (10) Na_2_HPO_4_, pH 7.6).

### FACS and microarrays

Acute cortical slices were prepared as described above and under a fluorescence dissecting scope (Leica, M165 FC), the E14.5 pallium including the SVZ stream and the E18.5 and P2.5 developing cortices excluding the SVZ stream were isolated from *GAD65-*GFP+ caudal coronal slices. Each time point was obtained in triplicate from three different litters and consisted of pooled embryos or pups (*n*=4–5). Following dissociation of the tissue, *GAD65*-GFP^+^ cells were isolated using FACS with the FACS VantageSE and collected in RNA Later solution (Invitrogen). RNA from the cells was extracted using the RNeasy Mini kit (Qiagen) and the quality was checked on an Agilent 2100 Bioanalyser (Agilent Technologies). A small-scale protocol from Affymetrix (High Wycombe) was used to reproducibly amplify and label total RNA. Briefly, approximately 100 ng total RNA was converted into double-stranded cDNA using a cDNA synthesis kit (Superscript; Invitrogen) with a special oligo(dT)_24 primer containing a T7 RNA promoter site added to the 5′ poly(T) tract. After the first cRNA amplification by *in vitro* transcription using the Ambion MEGAscript T7 kit (Ambion), 400 ng cRNA was once more reverse transcribed, and biotinylated cRNAs were generated from double-strand cDNAs using an *in vitro* transcription labelling kit from Affymetrix. For each probe, 20 μg of the second amplification biotinylated cRNA was fragmented and hybridized to Mouse Genome 430 2.0 Array (Affymetrix) following the standard protocols. GeneChips were incubated at 45 °C for 16 h with biotin-labelled cRNAs probes, and washed and stained using a streptavidin–phycoerythrin conjugate with antibody amplification using Affymetrix GeneChip Fluidis Station 450. GeneChips were scanned on a GC3000 scanner (Affymetrix). The signal intensities were analysed using the Partek Genomics suites (version 6.6 beta) and the data were normalized using robust multi-array average (RMA)^59^. The messenger RNA expression levels of genes (ProbeSet) were compared using a two-way analysis of variance model and contrasted with uncorrected *P* values set at <0.01 threshold. Selection of target genes specifically increasing their expression during CP invasion at E18.5 was based on >2-fold intensity increase from E14.5 versus E18.5 and >2-fold intensity decrease from P2.5 versus E18.5.

### ELISA quantification of protein expression

Microdissection of the CGE was performed at E14.5, trypsinized dissociated cells were plated on 96-well plates and cultured in NMB supplemented with *m*CPBG (10 μM; Tocris) and the mitotic inhibitor arabinofuranosyl cytidine (1 μM). Quantification of protein levels was performed using ELISA kit (Thermo scientific) and 5-HT_3A_R antibody (1:1,000, LSBio) on E14.5 day *in vitro* 1 (+DIV1) or E14.5 (+DIV4) cells. Absorbance levels were measured on a SpectraMax Paradigm Multimode Microplate detection reader (Molecular Devices). Absorbance levels for the 5HT_3A_R were normalized to absorbance levels calculated for the total amount of cells via Janus Green whole-cell staining.

### Focal electroporation on slices

For focal electroporation on slices, acute coronal slices of E14.5 *Htr3a*-GFP^+^ embryos were transferred on membranes (nucleopore Track-Etch; 1 μm pore size, Whatman) floating on NBM in an incubator (37 °C, 5% CO_2_) for at least 2 h following Vibratome sectioning. A plasmid encoding for a red fluorescent protein (pUBI-tdTOM) was focally injected at the concentration of 1 μg ml^−1^ through a beveled glass micropipette into the MGE using a nanoinjector (B203XVZ; WPI) and precision of the injection site was controlled by addition of 10% Fast Green (Sigma) to the plasmid. The slices were kept moisturized in HBSS and electroporated between platinum Petri dish and cover square platinum electrodes (CUY701-P5E and CUY701-P5L; Nepa Gene) with two unipolar square pulses (Δ*P*: 100 V; duration: 5 ms; interval: 500 ms) generated by a CUY21-SQ electroporator (Nepa Gene). Slices were then cultured 3 days *in vitro* (+DIV3) in an incubator (37 °C and 5% CO_2_).

### Calcium imaging and analysis

Calcium experiments were performed in CGE-isolated cultures (E14.5 +DIV1–3; see above; Preparation of dissociated cultures and acute slices). Cell cultures were loaded with Fura-2AM (1 μM; Invitrogen) in NBM for 20 min in an incubator (37 °C, 5% CO_2_). Observations were done in a bath chamber (RC-26; Warner Instruments) continuously superfused with ACSF (95% O_2_, 5% CO_2_) under an inverted epifluorescence microscope (Observer Z1; Zeiss) equipped for live imaging (Life Technologies) with an oil-immersion × 40 objective (Zeiss, Fluar × 40/1.3 oil Ph3) and CoolSNAP-HQ camera. ACSF and microscope bath chamber temperatures were kept at 37 °C. The software MetaFluor (version 7.7.0.0; Molecular Devices) was used to acquire images each second and monitor intracellular calcium changes. Calcium events were analysed in Clampfit (version 9.2.0.11; Molecular Devices). A baseline recording was obtained before challenging cells with *m*CPBG (1 μM,) or SR57227 hydrochloride (1 μM). To isolate specific responses to 5-HT_3A_R activation, experiments were performed with blockers for voltage-gated sodium channels, AMPA receptors, NMDA receptors and GABA_A_ receptors (respectively, TTX, 200 nM; NBQX, 2 μM; D-AP5, 20 μM and gabazine, 4 μM; Tocris) superfused 5 min before and along with *m*CPBG. Cell viability was systematically assessed by a final surperfusion of 100 mM KCl in ACSF. A calcium event was defined as a calcium increase two times greater than the baseline activity and lasting for more than 1 s. *Htr3a*-GFP^+^ cINs were recorded at E14.5 (+DIV1) and E14.5 (+DIV3) in the presence and absence of blockers.

### Electrophysiological recordings

Coronal brain sections of 300-μm thick were obtained at E14-E15, E18-E19, P2/P3 using a Vibratome (Leica, VT1000S). During recording, slices were continuously superfused with ACSF containing (in mM): NaCl (120), KCl (3.5), CaCl_2_ (2.5), MgSO_4_ (1.3), NaH_2_PO_4_ (1.25), NaHCO_3_ (25), glucose (25), continuously bubbled with 95% O_2_ and 5% CO_2_, pH 7.4. Whole-cell voltage and current clamp recordings were made from visually identified GFP^+^ neurons using an EPC9 amplifier (HEKA) and an upright microscope (Zeiss Axioskop FS2) equipped with infrared differential interference contrast (IR-DIC) and standard epifluorescence. Patch pipettes had a resistance of 4–6 MΩ when filled with (in mM): 105 K-gluconate, 30 KCl, 0.5 CaCl_2_, 5 EGTA, 2 Mg-ATP, 10 HEPES. Application of serotonin (5-HT, 100 μM; Tocris) or SR 57227 hydrochloride (100 μM; Tocris) was performed via pressure ejection from a second pipette connected to a picospritzer. To isolate specific responses to 5-HT_3A_R activation, recordings at E17-E18 were performed with blockers (TTX, 1 μM; NBQX, 10 μM; D-AP5, 100 μM and gabazine, 20 μM; Tocris). Cells were voltage clamped at −60 mV and currents were evoked by series of 20-ms-duration depolarizing voltage pulses. Current clamp recordings were performed in response to long (1 s) hyperpolarizing and depolarizing current injections. Signals were filtered at 1–5 kHz, sampled at 2–10 kHz and off-line analysis was performed using Igor Pro (Wavemetrics).

### Time-lapse imaging and analysis

Time-lapse imaging of migrating *GAD65*-GFP^+^ cells in cultures was performed using an automated microscope (ImageXpress Micro XL; Molecular Devices) equipped with objective × 10 (Plan Fluor × 10/0.30 Ph1), which allows the simultaneous recording of migrating cells in multiple wells at 37 °C under 95% O_2_ and 5% CO_2_. Following a control time-lapse imaging period of 360 min, SR57227 hydrochloride (100 nM; Tocris), serotonin (5-HT, 100 nM; Tocris) or vehicle (NBM) was added to the medium and recordings were performed for an additional 360 min. Using Metamorph software (version 7.4; Molecular Devices), cells were tracked at E14.5 (+DIV1) and at E17.5 (+DIV1). Time-lapse imaging of cortical invasion of cINs was performed on cortical slices at E17.5, placed on porous nitrocellulose (Millicell-CM, Millipore) inserts in Fluorodishes (WPI) filled with NBM and with an inverted confocal microscope (Nikon A1R) equipped for live imaging (Life Technologies) with a long working distance × 20 objective (CFI Plan Fluor ELWD C × 20/0.45, Nikon). The microscope incubation chamber temperature was kept at 37 °C with a constant flux (25 l h^−1^) of 5% CO_2_ humidified at 96%. 50-μm-thick stacks (3 μm-stepped) were acquired every 10 min during 10–12 h, with resonant laser scanning to reduce toxicity and to avoid bleaching. The first 90 min of movies were removed from analysis to avoid bias in measurements because of settling of the slices in the microscope chamber. For quantification of cortical invasion, the intermediate zone (260–410 μm from the pia) and cortical (50–220 μm from the pia) compartments were manually drawn in Metamorph and GFP^+^ INs from *GAD65*-GFP^+^ and *Htr3a*-ko*; GAD65*-GFP^+^ slices located in the IZ compartment at start were tracked during 8 h. The percentage of *GAD65*-GFP^+^ INs entering the cortex and not going back into the IZ or further migrating in the MZ during the 8-h time period was calculated. Migration speed was calculated as the total distance travelled by *GAD65*-GFP^+^ INs divided by total imaging time excluding the pausing time. The percentage pausing time was calculated as the fraction of time the cell nucleus was stationary during the imaging period. For simultaneous imaging of growth cones of CGE INs and MGE INs, E14.5 (+DIV3) *Htr3a*-GFP^+^ slices that were focally electroporated in the MGE (see Focal electroporation on slice) were placed in the bath chamber (RC-26; Warner Instruments) and were immobilized with an anchor. Imaging was done in the CP with a continuous superfusion of oxygenized ACSF (95% O_2_, 5% CO_2_) under an inverted confocal microscope (Nikon, A1R) equipped for live imaging with a × 60 oil-immersion objective (Plan Apo VC H × 60/1.4, Nikon) with a × 3 numerical zoom. ACSF and microscope incubation chamber temperatures were kept at 37 °C. 10-μm-thick stacks (1 μm-stepped) were acquired every 2 min with resonant laser scanning. Dual imaging was done in channel series mode to avoid fluorescence crosstalk. Imaging sessions were systematically started 20–30 min after immersion of the slice in ACSF to avoid axial drift, loss of focus and bias in measurement because of settling of the slice. A standard imaging session was composed of a baseline period of 14 min followed by 14 min drug application (*m*CPBG, 100 μM; Tocris) and 2 × 14 min wash. Time-lapse stacks were aligned using Metamorph software (version 7.4). For quantification, contours of the growth cone were manually drawn using Metamorph and their speed of progression as well as their area was measured. The speed was calculated by tracking the base of the growth cone. The leading process was taken as an axis of growth so that all movements towards the growth cone were considered positive and those towards the cell body negative.

### *In vivo* grafts

The CGE of E14.5 *GAD65*-GFP^+^ or *Htr3a*-ko; *GAD65*-GFP^+^ mice was isolated, cells were dissociated and resuspended to obtain a cell suspension of approximately 100,000 cells per μl. 10% Fast Green (Sigma) was added to the cell suspension as a tracer. Using a nanoinjector (WPI, B203XVZ), approximately 1 μl of the cell suspension was injected into the CGE of embryos from pregnant E14.5 WT using beveled glass pipettes with a diameter of 70–100 μm. Embryos from host mouse were then collected at E19 for tissue processing and analysis. Only embryos with grafted cells observed in the CGE at E19 were used for the quantification.

### Quantification of IN identity and distribution

Images were acquired using an epifluorescence microscope (Nikon, Eclipse 90i) equipped with a × 10 objective (Plan Apo × 10/1, Nikon) or a confocal (Nikon, A1R) microscope equipped with dry × 10 and × 20 objectives (CFI Plan Apo × 10/0.45 and CFI Plan Apo VC; × 20/0.75, Nikon) and oil-immersion × 40 and × 60 objectives (CFI Plan Fluor × 40/1.3 and CFI Plan Apo VC H × 60/1.4, Nikon). Quantification of the distribution of IN subtypes in the cortex was done by apposing a 10-bin grid at the level of the primary somatosensory cortex and bins corresponding to cortical layers were pooled.

### Statistical analysis

No statistics were used to determine optimal group sample size; however, sample sizes were similar to those used in previous publications from our group and others. No samples were excluded from statistical analysis. Statistical analyses (GraphPad Prism software, version 6.0) were performed using paired or unpaired Student’s *t*-test, one-way or two-way analysis of variance with Tukey’s or Bonferroni’s multiple comparisons test.

## Author contributions

A.D. and T.V. conceived the project, A.D., T.V., P.C. and JAvH designed the experiments and S.M., M.N., N.H., G.L., S.F., P.C. and JAvH performed the experiments. A.D. and T.V. wrote the manuscript.

## Additional information

**How to cite this article**: Murthy, S. *et al.* Serotonin receptor 3A controls interneuron migration into the neocortex. *Nat. Commun.* 5:5524 doi: 10.1038/ncomms6524 (2014).

## Supplementary Material

Supplementary Information Supplementary Figures 1-5, Supplementary Tables 1-2 and Supplementary References

Supplementary Movie 1 Time-lapse sequence showing that the 5-HT_3A_R agonist (SR57227 100 nM) stimulates the migration of E17.5 (+DIV1) *GAD65*-GFP+ interneurons (INs). End image of the movie depicts examples of migratory tracks of *GAD65*-GFP+ INs during the control period (in white) that are shorter in distance compared to the SR57227 condition (in red). Scale bar: 10 μm.

Supplementary Movie 2 Time-lapse sequence showing that the 5-HT_3A_R agonist (SR57227 100 nM) does not stimulate the migration of E14.5 (+DIV1) *GAD65*-GFP+ INs. End image of the movie depicts examples of migratory tracks of *GAD65*-GFP+ INs during the control period (in white) that are not increased in distance compared to the SR57227 condition (in red). Scale bar: 10 μm.

Supplementary Movie 3 Time-lapse sequence showing that 5-HT_3A_R agonist (*m*CPBG 100μM) exposure (red arrows) induces a delayed increase in the growth cone (G.C.) size of a *Htr3a*-GFP+ interneuron migrating in the cortical plate. End image of the movie depicts the travel and examples of growth cone areas during baseline (white), 5-HT*3A*R agonist exposure (red), wash 1 (green) and wash 2 (cyan). Scale bar: 5 μm.

Supplementary Movie 4 Time-lapse sequence showing that 5-HT_3A_R agonist (*m*CPBG 100μM) exposure (red arrows) does not modify the growth cone (G.C.) size of a TOM+ labeled MGE-derived interneuron migrating in the cortical plate. End image of the movie depicts the travel and examples of growth cone areas during baseline (white), 5-HT_3A_R agonist exposure (red), wash 1 (green) and wash 2 (cyan). Scale bar: 5 μm.

Supplementary Movie 5 Time-lapse sequence showing E17.5 *GAD65*-GFP+ INs migrating from the intermediate zone (IZ) (white arrowheads) into the cortical plate (CP) (red arrowheads). A higher proportion of *GAD65*-GFP+ INs enter the cortical plate (CP) (red arrowheads) compared to Htr3a-ko; *GAD65*-GFP+ INs (Supplementary movie 6). End image of the movie depicts examples of migratory tracks of *GAD65*-GFP+ INs migrating into the CP (in red) or staying in the IZ (in white). Scale bar: 50 μm.

Supplementary Movie 6 Time-lapse sequence showing *Htr3a*-ko; *GAD65*-GFP+ INs preferentially remaining in the E17.5 intermediate zone (IZ) (white arrowheads). A higher proportion of *Htr3a*-ko; *GAD65*-GFP+ INs remain in IZ (white arrowheads) compared to GAD65-GFP+ INs (Supplementary movie 5). End image of the movie depicts examples of migratory tracks of *Htr3a*-ko; *GAD65-GFP+* INs remaining in the IZ (in white). Scale bar: 50 μm.
