# Supplementary material for: Serotonin receptor 3A controls interneuron migration into the neocortex
Source: Nat Commun. 2014 Nov 20;5:5524. doi: 10.1038/ncomms6524 (PMC4263148; doi:10.1038/ncomms6524)
Supplement: Supplementary Information — Supplementary Figures 1-5, Supplementary Tables 1-2 and Supplementary References [file ncomms6524-s1.pdf]

Title: Serotonin receptor 3A controls interneuron migration into the neocortex

Sahana Murthy, Mathieu Niquille, Nicolas Hurni, Greta Limoni, Sarah Frazer, Pascal Chameau, Johannes A. van Hooft, Tania Vitalis, Alexandre Dayer

Supplementary Figures

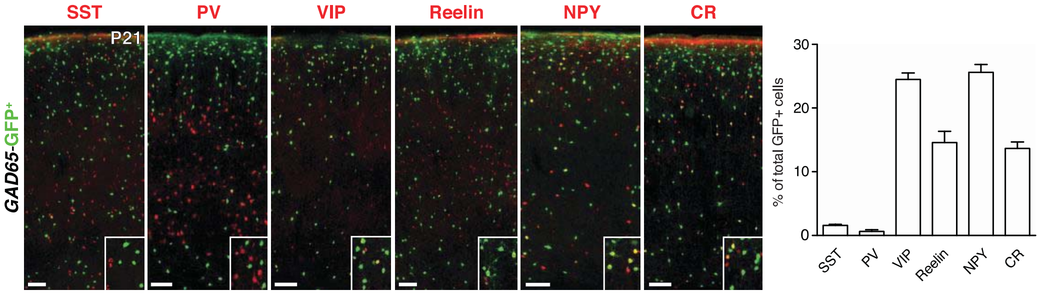

Supplementary Figure 1. *GAD65*-GFP mice mainly label CGE-derived cortical interneurons. At P21 *GAD65*-GFP+ INs hardly express MGE-derived markers (< 5%) (see insets) such as parvalbumin (PV; n = 999 cells in 5 brains) and somatostatin (SST; n = 748 cells in 3 brains). By contrast, *GAD65*-GFP+ INs were found to express CGE-derived markers (see insets) such as vasointestinal peptide (VIP; n = 1091 cells in 5 brains), reelin (n = 1376 in 6 brains), neuropeptide Y (NPY; n = 1127 in 5 brains) and calretinin (CR; n = 1577 in 6 brains). Error bars are means ± SEM. Scale bar: 100 µm.

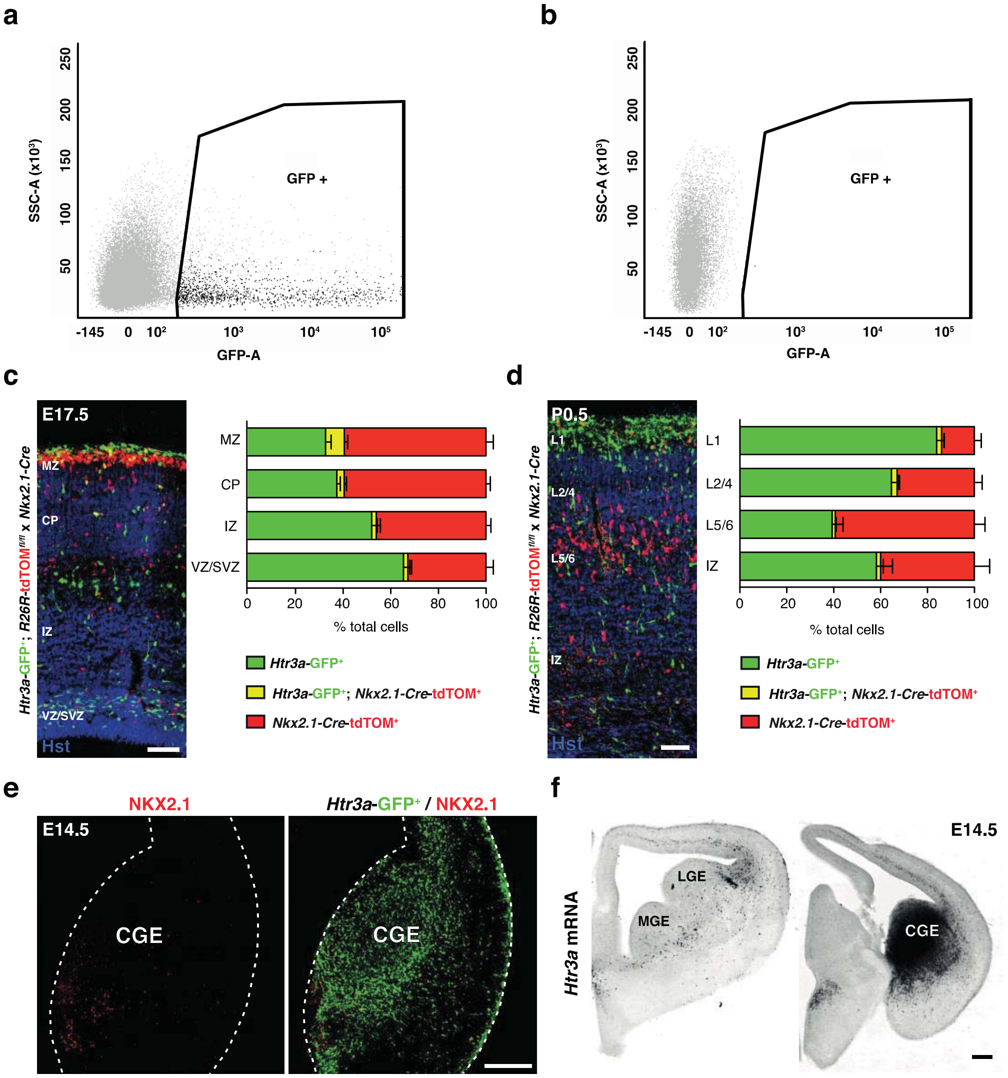

Supplementary Figure 2. The 5-HT$_{3A}$R is specifically expressed in CGE-derived cortical interneurons. Microdissection of *GAD65*-GFP+ cortices was performed at 3 developmental time-points (See Fig.1) and *GAD65*-GFP+ interneurons (INs) were isolated using fluorescence-activated cell sorting (FACS). (a, b) Representative profiles of FACS-isolated *GAD65*-GFP+ INs (a) and *GAD65*-GFP- cells (b) at E18.5. X-axis represents GFP intensity in arbitrary units and y-axis represents side-scatter in arbitrary units. (c, d) Genetic fate mapping indicates that *Htr3a*-GFP+ INs rarely overlap with *Nkx2.1*-Cre-tdTOM+ INs at E17.5 (c; n =1587 GFP+ cells and 1602 TOM+ cells in 2 brains) and P0.5 (d; n = 1270 GFP+ cells and 874 TOM+ cells in 2 brains). Note at P0.5 a preferential distribution of *Htr3a*-GFP+ INs in prospective superficial layers 1-4 in contrast to *Nkx2.1*-Cre-tdTOM+ INs that are preferentially targeted to deep cortical layers 5/6. (e) Immunohistochemistry showing that the vast majority of *Htr3a*-GFP+ INs in the CGE do not express the MGE-specific factor NKX2.1. (f) *In situ* hybridization showing strong expression of the *Htr3a* mRNA in the E14.5 CGE but not in the MGE. Error bars are means ± SEM. CGE: caudal ganglionic eminence, CP: cortical plate, Hst: Hoechst, IZ: intermediate zone, LGE: lateral ganglionic eminence, MGE: medial ganglionic eminence, MZ: marginal zone, SVZ: subventricular zone, VZ: ventricular zone.. Scale bars: (c, d) 100 µm; (e, f) 200 µm.

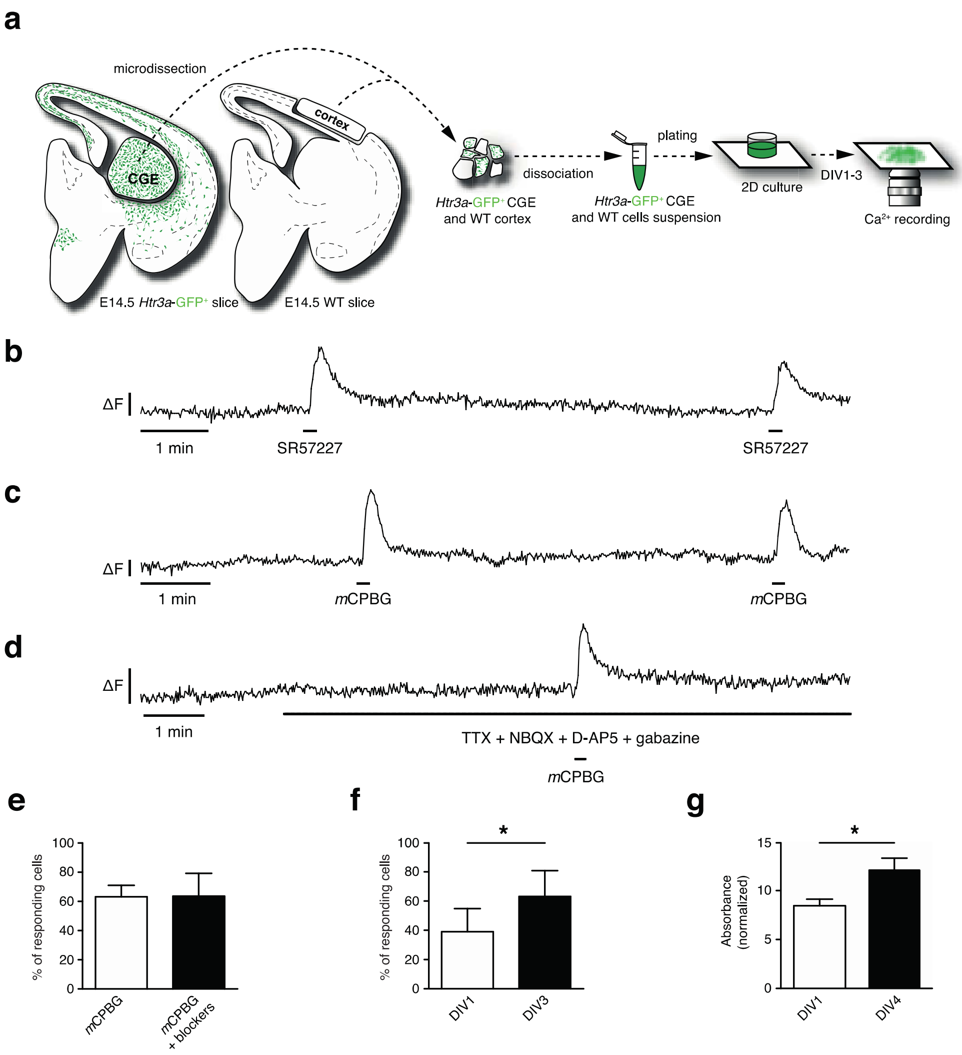

Supplementary Figure 3. 5-HT$_{3A}$R activation triggers calcium transients in migrating CGE-derived cortical interneurons. (a) The E14.5 caudal ganglionic eminence (CGE) of *Htr3a*-GFP+ slices was isolated by microdissection and platted *in vitro* with dissociated cortical tissue to allow *Htr3a*-GFP+ INs to migrate on a cortical cellular substrate. Calcium imaging was performed at day in vitro 1 (+DIV1) or (+DIV3) on migrating INs. (b, c) Illustrative calcium traces from *Htr3a*-GFP+ cells at E14.5 (+DIV3) showing that the 5-HT$_{3A}$R agonists SR57227 (1 µM; b) and *m*CPBG (1 µM; c) both trigger calcium transients. (d) Illustrative calcium trace from a *Htr3a*-GFP+ cell at E14.5 (+DIV3) showing that 5-HT$_{3A}$R activation (*m*CPBG; 1 µM) triggers a calcium transient in the presence of TTX (200 nM), NBQX (2 µM), D-AP5 (20 µM) and gabazine (4 µM). (e) Graph showing that the percentage of *Htr3a*-GFP+ responding to *m*CPBG is not different (unpaired Student's t-test) with (n = 28 cells) or without TTX, NBQX, D-AP5, gabazine (n = 89 cells) (f) Graph showing that the percentage of *Htr3a*-GFP+ responding to *m*CPBG significantly increases from E14.5 (+DIV1) (n = 62 cells) to E14.5 (+DIV3) (n = 89 cells) (* $P < 0.05$, unpaired Student's t-test) (g) Graph showing that protein expression of the 5-HT$_{3A}$R significantly increases from E14.5 (+DIV1) to E14.5 (+DIV4) (* $P < 0.05$, unpaired Student's t-test). Error bars are means ± SEM of a least 3 replicates for each experiment.

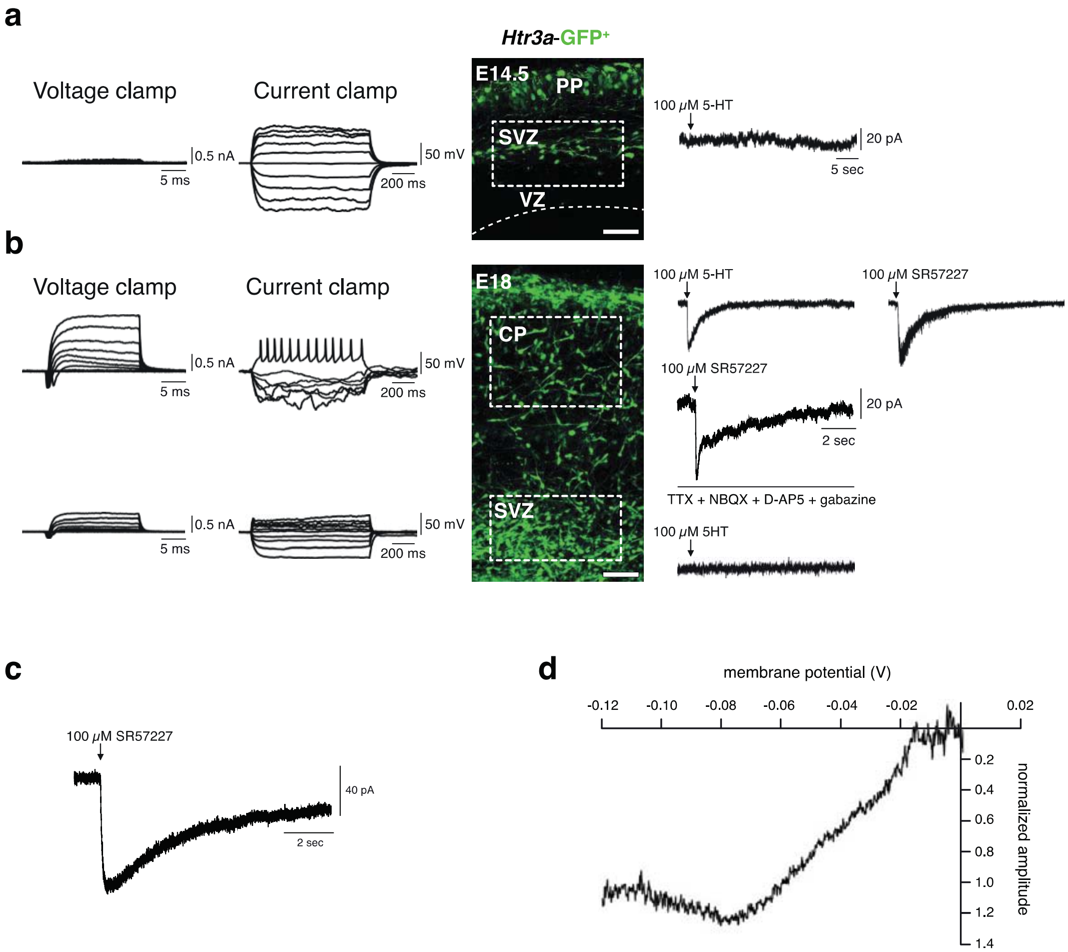

Supplementary Figure 4. 5-HT$_{3A}$R activation triggers inward currents in migrating CGE-derived cortical interneurons. 5-HT$_{3A}$R activation induces inwards currents in *Htr3a*-GFP+ cINs migrating in the cortical plate (CP) but not in the subventricular zone (SVZ) / intermediate zone (IZ) during the process of tangential migration. In the SVZ / IZ at E14.5 (a), in the SVZ at E18 (b bottom), *Htr3a*-GFP+ cells display no action potentials and small inward Na^+ currents. Only *Htr3a*-GFP+ cells located in the E18 CP (b top) respond with a 5-HT$_{3A}$R-mediated inward current upon application of 100 µM serotonin (5-HT) or 100 µM SR57227 in absence (b top) or presence (b middle) of TTX, NBQX, D-AP5 and gabazine. (c, d) At P2.5 application of 100 µM SR57227 consistently induced 5-HT$_{3A}$R-mediated inward currents (c) which display the characteristic region of negative slope conductance in the I-V curve[1,2] (d). Voltage-dependent currents were evoked under voltage clamp by 10 steps of 10 mV from -60 mV. Voltage responses under current clamp were evoked by 10 steps of 20 pA from -100 pA. I-V curves were recorded by applying a voltage ramp from 0 mV to -120 mV during the 5-HT$_{3A}$R-mediated inward current as previously described[1,2]. PP: preplate. VZ: ventricular zone. Scale bars: (a) 50 µm; (b) 100 µm.

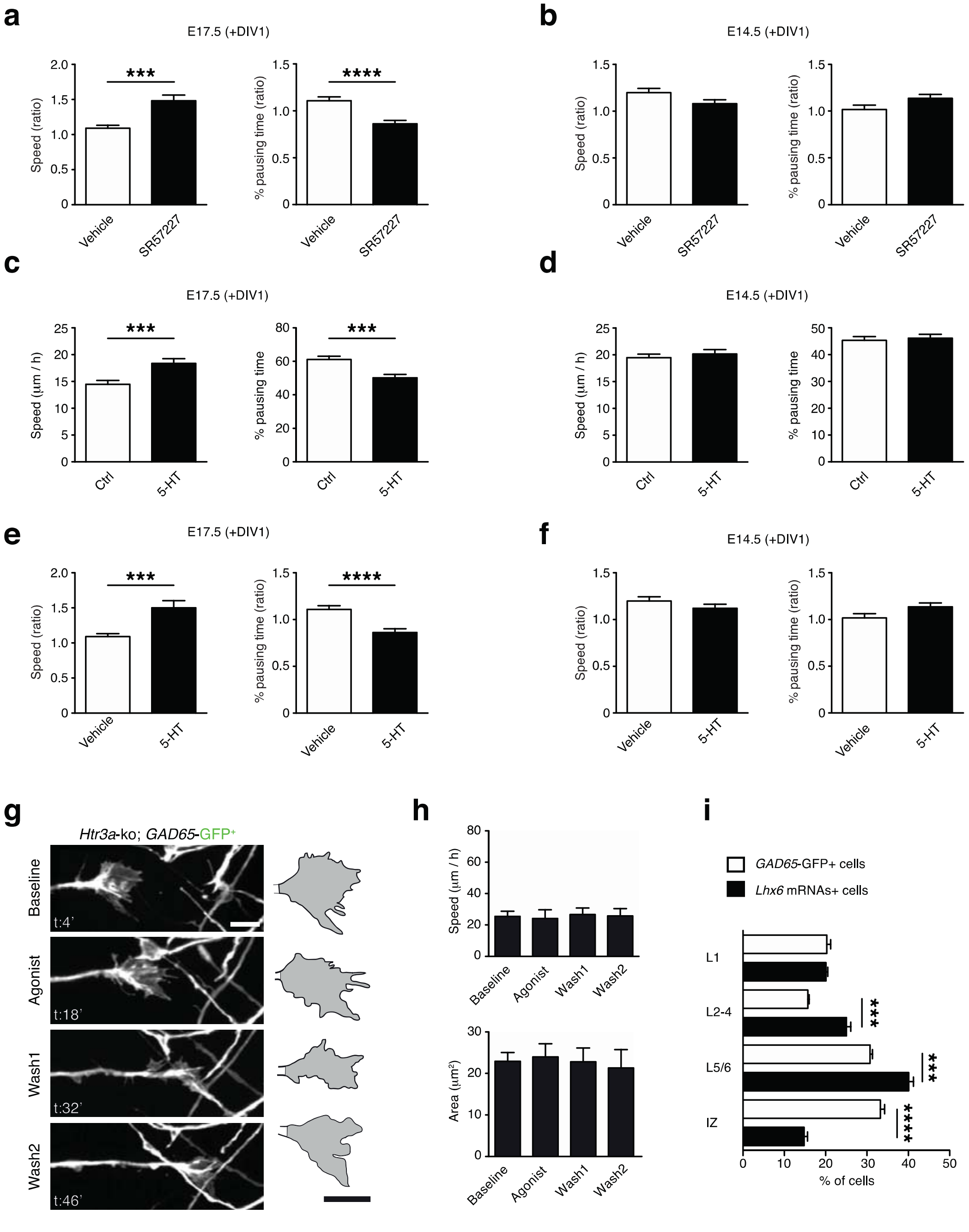

Supplementary Figure 5. 5-HT$_{3A}$R activation stimulates the migration of CGE-derived cortical interneurons. In cell culture time lapse imaging (see Fig. 2), *GAD65*-GFP+ cells were tracked during a control period and during a drug application period conducted either with a vehicle (NBM) or with a 5-HT$_{3A}$R agonist (5HT or SR57227; 100 nM). Each period consisted of 360 min recording and at least three independent experiments were performed for each time-point. (a, b) Speed and percentage pausing time ratios following SR57227 application are significantly modified compared to a vehicle control condition at E17.5 (+DIV1) (n = 125 cells vehicle, n = 156 cells SR57227) (*** $P < 0.001$, **** $P < 0.0001$, unpaired Student's t-test) (a) but not at E14.5 (+DIV1) (n = 114 cells vehicle, n = 141 cells SR57227) (b) (c, d) Application of serotonin (100 nM) significantly increases the migratory speed and significantly

decreases the pausing time of *GAD65*-GFP+ interneurons at E17.5 (+DIV1) (n = 110 cells) (*** *P* < 0.001, paired Student's t-test) (c) but not at E14.5 (+DIV1) (n = 170 cells) (d). (e, f) Speed and percentage of pausing time ratios following serotonin application are significantly modified compared to a vehicle control condition at E17.5 (+DIV1) (n = 125 cells vehicle, n = 110 cells serotonin) (*** *P* < 0.001, **** *P* < 0.0001, unpaired Student's t-test) (e) but not at E14.5 (+DIV1) (n = 114 cells vehicle, n = 170 cells serotonin) (f). (g) Time-lapse sequence illustrating that 5-HT_{3A}R activation (*m*CPBG; 100µM) fails to induces a delayed increase in the growth cone (GC) size of *Htr3a*-ko*;* *GAD65*-GFP+ growth cones (h) Quantification revealed that the GC speed and size of *Htr3a*-ko; *GAD65*-GFP+ INs (n = 10 GC in 4 slices) is not significantly modified after 5-HT_{3A}R activation (one-way ANOVA with Bonferroni's test). (i) Quantification at P0.5 reveals significant differences in the percentage of *GAD65*-GFP+ INs (n = 6708 cells in 4 brains) in layer 2-4, layer 5/6 and intermediate zone (IZ) (*** *P* < 0.001, **** *P* < 0.0001, unpaired Student's t-test) compared to MGE-derived INs labeled by *in situ* hybridization for the MGE-specific transcription factor *Lhx6* (n = 3731 cells in 4 brains). Error bars are means ± SEM Scale bar: (g) 5 µm.

Supplementary Table 1: Genes transiently up-regulated in *GAD65*-GFP+ interneurons during cortical invasion at E18.5

Gene Symbol	Gene Title	Fold-Change (E18.5 vs. E14.5)	p-value	Fold-Change (P2.5 vs. P18.5)	p-value
Ntf3	Neurotrophin 3	6.34	0.0001662	-4.77	0.0006681
Ccbe1	Collagen and calcium binding EGF domains 1	6.33	0.0005796	-3.43	0.0092652
Htr3a	**5-hydroxytryptamine (serotonin) receptor 3A**	**4.90**	**0.0000034**	**-2.93**	**0.0001453**
Nr4a3	Nuclear receptor subfamily 4, group A, member 3	4.05	0.0000197	-2.04	0.0048343
Glt28d2	Glycosyltransferase 28 domain containing 2	4.03	0.0000268	-2.91	0.0003009
Adamts2	A disintegrin-like and metallopeptidase (reprolysin type) with thrombospondin type 1	4.02	0.0000262	-2.37	0.0015290
Ccno	Cyclin O	3.98	0.0000790	-2.78	0.0009906
Cacna2d1	Calcium channel, voltage-dependent, alpha2/delta subunit1	3.80	0.0000913	-3.26	0.0002670
Hs6st2	Heparan sulfate 6-O-sulfotransferase 2	2.90	0.0030536	-3.98	0.0004241
Ryr1	Ryanodine receptor 1, skeletal muscle	2.85	0.0012057	-2.28	0.0061242
Dlg2	Discs, large homolog 2 (Drosophila)	2.39	0.0032731	-2.40	0.0031401
Ndrg1	N-myc downstream regulated gene 1	2.22	0.0071545	-2.33	0.0049528
Plxna4	Plexin A4	2.20	0.0005265	-2.36	0.0002516
Ptpro	Protein tyrosine phosphatase, receptor type, O	2.05	0.0096454	-2.09	0.0081087

Legend: List of genes displaying a significant up-regulation from E14.5 to E18.5 (> 2-fold increase, $p < 0.01$, two-way ANOVA with Fisher's Least Significance Difference) and a significant down-regulation from E18.5 to P2.5 (> 2 fold decrease, $p < 0.01$, two-way ANOVA with Fisher's Least Significance Difference) in FACS-isolated *GAD65*-GFP+ cortical interneurons.

Supplementary Table 2 : 5-HT$_{3A}$R-mediated inward currents and electrical properties of *Htr3a*-GFP+ interneurons

Age	Location	Response to 5-HT or SR (pA)	Capacitance (pF)	Input resistance (GΩ)	Resting membrane potential (mV)	Number of cells firing AP	n = cells
E14-E15	SVZ	0	4.9 ± 0.3	2.0 ± 0.3	-17.5 ± 4.0	0	8
E18-E19	SVZ	0	8.1 ± 0.9	1.8 ± 0.3	-31.9 ± 3.6	0	8
	CP	5-HT: -20.9 ± 4.0 (n=5/7)	6.8 ± 0.7	2.1 ± 0.4	-39.7 ± 2.1	2	18
		SR: -46.4 ± 21.4 (n=8/11)					
P2-P3	CP	5-HT: -83.3 ± 24.2 (n=6/6)	10.1 ± 0.6	1.2 ± 0.2	-52.5 ± 3.4	10	12
		SR: -71.4 ± 32.6 (n=6/6)					

Legend: E14-E15 *Htr3a*-GFP+ migrating interneurons (INs) located in the SVZ at E14-E15 and E18-E19 *Htr3a*-GFP+ INs display no action potential and are not responsive to 5-HT application (n = 0 out of 8 in each conditions). Rare E18-E19 *Htr3a*-GFP+ INs located in the CP (n = 2 out of 18) display action potentials but most of them respond to 100 µM 5-HT (n = 5 out of 7) or to 100 µM SR 57227 application (n = 8 out of 11) in voltage clamp (holding potential: -70 mV). At P2-P3, all 5-HT$_{3A}$+ cells respond to the application of either 5-HT or SR 57227, and the majority fire at least one action potential upon injection of +150 pA in current clamp mode (n = 10 out of 12). Numbers are means ± SEM.

Supplementary references.

1. Van Hooft, J. A. & Wadman, W. J. Ca2+ ions block and permeate serotonin 5-HT3 receptor channels in rat hippocampal interneurons. *J Neurophysiol* **89**, 1864-1869 (2003).

2. Noam, Y., Wadman, W. J. & van Hooft, J. A. On the voltage-dependent Ca2+ block of serotonin 5-HT3 receptors: a critical role of intracellular phosphates. *J Physiol* **586**, 3629-3638 (2008).
